# Evaluating willingness for surgery using the *SMART Choice (Knee)* patient prognostic tool for total knee arthroplasty: study protocol for a pragmatic randomised controlled trial

**DOI:** 10.1186/s12891-022-05123-0

**Published:** 2022-02-24

**Authors:** Yuxuan Zhou, Claire Weeden, Lauren Patten, Michelle Dowsey, Samantha Bunzli, Peter Choong, Chris Schilling

**Affiliations:** Department of Surgery, The University of Melbourne, Melbourne, Australia

**Keywords:** Total knee arthroplasty, Osteoarthritis, Prognostic tool, Machine learning, Artificial intelligence, Predictive model, Decision support tools, Patient-reported outcome measures, Willingness for surgery, Patient satisfaction

## Abstract

**Background:**

Approximately 1 in 5 patients feel unsatisfied after total knee arthroplasty (TKA). Prognostic tools may aid in the patient selection process and reduce the proportion of patients who experience unsatisfactory surgery. This study uses the prognostic tool *SMART Choice (Patient Prognostic Tool for Total Knee Arthroplasty)* to predict patient improvement after TKA. The tool aims to be used by the patient without clinician input and does not require clinical data such as X-ray findings or blood results. The objective of this study is to evaluate the *SMART Choice* tool on patient decision making, particularly willingness for surgery. We hypothesise that the use of the *SMART Choice* tool will influence willingness to undergo surgery, especially when used earlier in the patient TKA journey.

**Methods:**

This is a multicentred, pragmatic, randomised controlled trial conducted in Melbourne, Australia. Participants will be recruited from the St. Vincent’s Hospital, Melbourne (SVHM) Orthopaedic Clinic, and the client base of HCF, Australia (private health insurance company). Patients over 45 years of age who have been diagnosed with knee osteoarthritis and considering TKA are eligible for participation. Participants will be randomised to either use the *SMART Choice* tool or treatment as usual. The *SMART Choice* tool provides users with a prediction for improvement or deterioration / no change after surgery based on utility score change calculated from the Veterans-RAND 12 (VR-12) survey. The primary outcome of the study is patient willingness for TKA surgery. The secondary outcomes include evaluating the optimal timing for tool use and using decision quality questionnaires to understand the patient experience when using the tool. Participants will be followed up for 6 months from the time of recruitment.

**Discussion:**

The *SMART Choice* tool has the potential to improve patient decision making for TKA. Although many prognostic tools have been developed for other areas of surgery, most are confined within academic bodies of work. This study will be one of the first to evaluate the impact of a prognostic tool on patient decision making using a prospective clinical trial, an important step in transitioning the tool for use in clinical practice.

**Trial registration:**

Australia and New Zealand Clinical Trials Registry (ANZCTR) - ACTRN12622000072718. Prospectively registered – 21 January 2022.

## Background

Knee osteoarthritis (OA) is a progressive and debilitating condition for sufferers. Pain and stiffness are common presenting complaints. Without adequate intervention, functional decline and even complete loss of independence can occur [1]. Lifestyle modification, analgesia and physiotherapy comprise the core of nonoperative management [2]. In certain situations, intra-articular injections may delay the need for surgery [3, 4]. Failing nonoperative management, the definitive treatment option for knee OA is total knee arthroplasty (TKA) [5].

Based on registry studies, TKA is generally regarded as a successful procedure [6, 7]. The risk of adverse events associated with surgery is relatively low, and the probability of improving symptoms is relatively high [8, 9]. However, recent studies have reported that up to 20% of patients remain unsatisfied after TKA [10, 11]. For these patients, ongoing symptoms from TKA severely impact their quality of life [12, 13]. With a current trend towards more arthroplasty surgeries globally, the social and economic impact of TKA dissatisfaction is a fast-growing problem [14].

To address this issue, solutions need to arise from multiple fronts. Improvements in surgical techniques and implant design seem to be the most obvious path forward. However, substantial progress has already been made from pioneers of the past. The trajectory of progress from technique and implant design alone is reaching a plateau [15, 16]. Furthermore, patients are dissatisfied despite what surgeons would perceive as successful surgery [17]. An alternative solution to TKA would be a completely new treatment for knee OA; a solution that addresses both the symptoms and natural history of the disease. Work is underway to experiment with biologic agents aimed at regenerating cartilage and bone [18–21]. However, this process is expensive and time consuming without any guarantee of success. Research must therefore explore complementary pathways to find solutions for TKA dissatisfaction.

One of these pathways is through improvement of patient-specific factors. The goal here is to optimise patients to become excellent surgical candidates. Prognostic tools fit into this area of research. These are tools developed to predict surgical outcomes. This is clinically useful in two ways. First, if poor outcomes can be predicted before surgery, then patients can be stratified into groups based on risk. For high-risk patients, resources can be set aside to improve modifiable risk factors. This may optimise the patients for surgery. Second, prognostic tools can manage patient expectations through informed decision making. A patient who understands their potential outcomes may regress their expectations towards what is realistic for their circumstances. This is based on the understanding that a major driver of dissatisfaction is the imbalance between expected and actual outcomes [22, 23]. The premise is that prognostic tools can better align these two perceptions to improve patient satisfaction and positively influence patient decision making around surgery.

The *SMART Choice* tool is a patient-focused prognostic tool that predicts clinical outcomes after TKA. The term “patient focused” means that a patient can use the tool without the input of a clinician. This allows the tool to be used early in the patient TKA journey. The *SMART Choice* tool was developed using data from the St. Vincent’s Melbourne Arthroplasty Outcomes (SMART) Registry– an extensive arthroplasty registry with over 14,000 patients and more than 20 years of follow-up time [24]. From this data, the *SMART Choice* tool can predict the chance of success. The tool defines success as an improvement in the health-related quality of life (HRQoL) utility score based on the previously calculated minimal clinically important difference (MCID) [25]. With the use of this tool, patients can gain benefits through two pathways: 1) informed decision making for the patient, and 2) managing patient expectations in preparation for TKA.

The rationale for this study is to evaluate the *SMART Choice* tool in a pragmatic clinical environment. We aim to investigate the influence of the *SMART Choice* tool on patient decision making and the optimal timepoint to use this tool in the patient TKA journey. Willingness for surgery is used as a proxy measurement for patient decision making because waiting for true occurrences of TKA would be unfeasible.

## Methods

This protocol is published in accordance with the SPIRIT (Standard Protocol Items for Randomised Trials) guidelines [26]. Furthermore, the *SMART Choice* tool was developed in accordance with TRIPOD (Transparent Reporting of Multivariable Prediction Model for Individual Prognosis or Diagnosis) Statement guidelines [27].

### SMART Choice tool development

This tool is being developed using a combination of traditional statistical methods and machine learning algorithms. Three thousand seven hundred fifty-five patients who underwent primary TKA procedures recorded on the SMART Registry between 2006 and 2019 will be analysed. Logistic regression, classification tree, XG boosted tree, and random forest models will be developed using sample splitting, 10-fold cross validation and bootstrapping techniques. Predictors considered include age at surgery, gender, and Veterans-RAND 12 (VR-12) responses. The model will predict a probability score (0–1) for improvement after TKA. From the probability score, participants will be stratified into deciles where the actual outcomes are reported as the output for the *SMART Choice* tool (Table 1).

**Table 1:** An example of probability score output from the *SMART Choice* tool development which correlates with a predicted outcome and actual outcome. The actual outcome within each decile will be reported to participants who use the tool. *Final probability scores will be determined once the SMART Choice tool predictive model is finalised *

Decile	Probability for Improvement (mean; range)	Predicted Outcome (n)	Actual Outcome (n; %)	
			Improvement	Deterioration/No Change
**1**	0.315 (0.119–0.402)	Deterioration/No Change (69)	24 (34.8)	45 (65.2)
**2**	0.456 (0.406–0.499)	Deterioration/No Change (69)	29 (42.0)	40 (58.0)
**3**	0.535 (0.501–0.564)	Deterioration/No Change (69)	29 (42.0)	40 (58.0)
**4**	0.586 (0.565–0.610)	Deterioration/No Change (69)	37 (53.6)	32 (46.4)
**5**	0.632 (0.610–0.651)	Deterioration/No Change (5)	1 (20.0)	4 (80.0)
		Improvement (63)	44 (69.8)	19 (30.2)
**6**	0.669 (0.652–0.688)	Improvement (68)	43 (63.2)	25 (36.8)
**7**	0.704 (0.688–0.725)	Improvement (68)	50 (73.5)	18 (26.5)
**8**	0.742 (0.726–0.764)	Improvement (68)	54 (79.4)	14 (20.6)
**9**	0.788 (0.764–0.812)	Improvement (68)	57 (83.8)	11 (16.2)
**10**	0.845 (0.812–0.928)	Improvement (68)	60 (88.2)	8 (11.8)

### Study design and setting

This is a prospective, pragmatic, assessor-blinded, superiority randomised controlled trial evaluating participants who have been diagnosed with knee OA and are considering TKA. Participants will be recruited from two sources: St. Vincent’s Hospital Melbourne (SVHM) Orthopaedic Outpatient Clinic and HCF (private health insurance company) client base across Australia.

From the SVHM source, patients who have been referred to an orthopaedic surgeon for consideration for TKA will be targeted for recruitment. This information can be found on clinic appointment lists in advance of 3 months. Patients who are referred to the clinic often wait up to 1 year to be seen by an orthopaedic surgeon. Following consultation, if deemed appropriate for surgery, patients may wait for another 6–12 months before the surgery is performed. Often, patients who are seen at SVHM are already in the later stages of the TKA journey as a product of resource constraints and waiting lists at the hospital.

In comparison, patients from the HCF source are at various stages of the TKA journey. As expected, some patients may be very early in disease progression, with symptoms being adequately managed by their general practitioner (GP). However, other patients may be more advanced in their disease progression and may have already made an application for TKA funding. Potential participants here will be recruited from internal advertising.

Having a diverse recruitment pool with patients at all stages of the TKA journey can help us evaluate the optimal timepoint for *SMART Choice* tool usage. We hypothesise that patients earlier in the TKA journey may be more open to decision making with consideration of predicted outcomes. In contrast, patients who are further along the TKA journey may feel too invested for the tool to influence decision making around willingness for surgery. The optimal timepoint for *SMART Choice* tool usage will be evaluated from a sub analysis of patients at different timepoints in the TKA journey.

### Eligibility criteria

Inclusion criteria will be selected for the following participants:

- Diagnosis of knee OA and consideration of primary unilateral TKA
- Considering primary and unilateral TKA
- Have already trialled nonoperative management for their knee symptoms
- Are willing and able to use web or mobile phone-based prognostic tool interfaces
- Able to provide informed consent to participate and available to be followed up for the duration of the study

The exclusion criteria were as follows:

- The source of knee symptoms are considered to be from any cause other than knee OA, e.g., rheumatoid arthritis, inflammatory arthritis, hip OA, referred lower back pain, etc.
- Are considering bilateral TKA, revision TKA, unicondylar knee arthroplasty, or patellofemoral arthroplasty
- TKA on the contralateral side
- Prior history of septic arthritis in the affected knee
- Significant bilateral knee symptoms
- Intra-articular injection in the affected knee within the last 3 months

### Randomisation, allocation, and blinding

Participants will be randomly allocated in a 1:1 ratio into two groups: intervention and treatment as usual (TAU). Allocation will be performed using a computer-generated simple randomisation schedule of consecutive patients. The randomisation will be embedded into the web-based platform that hosts the study portal. All participants who meet the eligibility criteria will be randomised.

Due to the nature of the study, participants will remain unblinded from their allocation group. However, limited disclosure of allocation groups will be applied to prevent participants who are allocated to TAU from proactively seeking out online prognostic tools to use. Investigators (excluding research assistants who will not be involved in data analysis) will remain blinded to the allocation group and identity of patients until after final data analysis is performed. Surgeons will be blinded to the allocation group of their patients and will have no influence on the outcome of allocation or intervention.

### Intervention and comparison groups

**Fig. 1:**
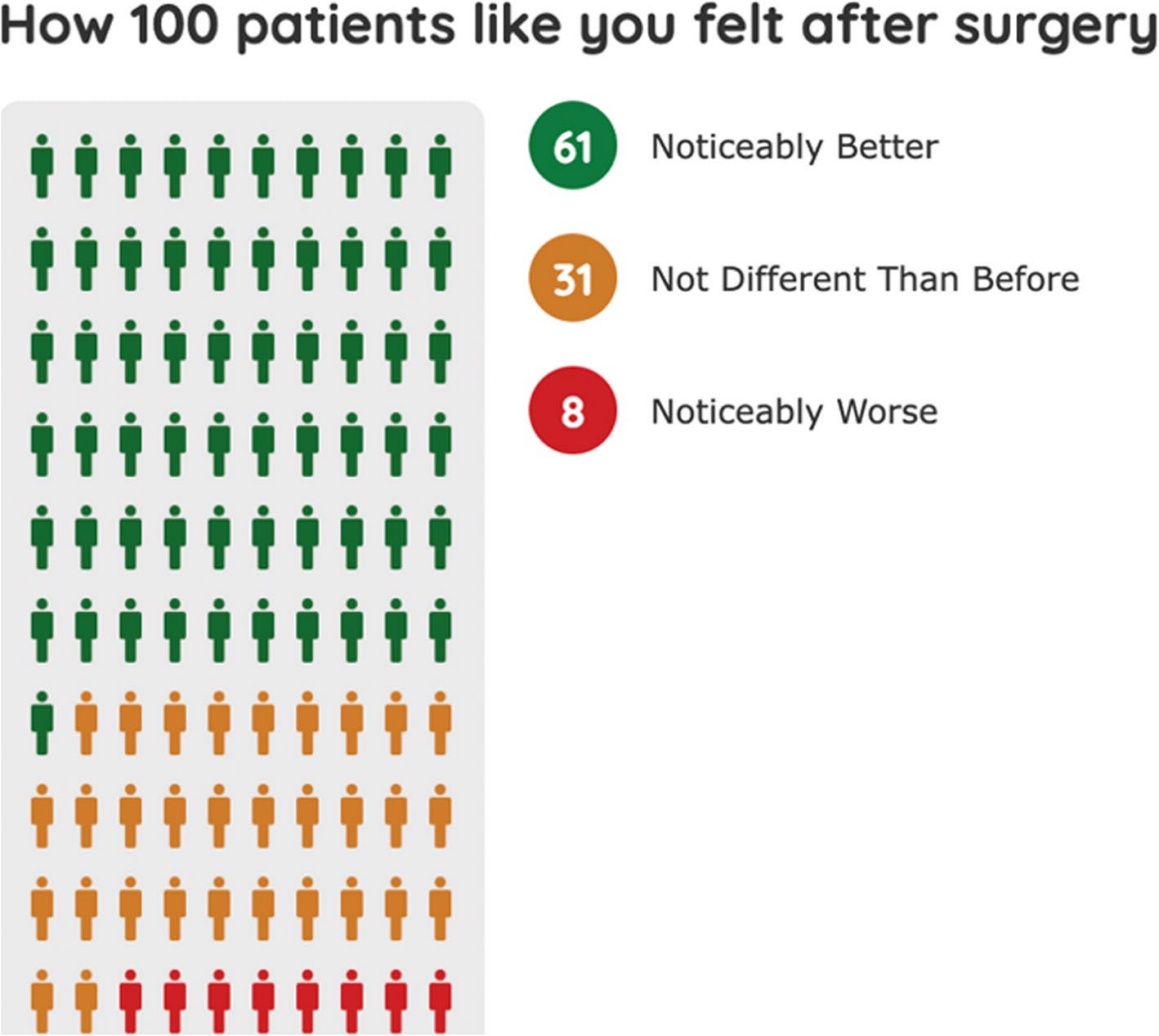
Screenshot of the *After My Surgery* tool for displaying predicted outcome after prognostic tool use. The *SMART Choice* tool will use a similar display format

Participants in the intervention group will receive use of the *SMART Choice* tool at the beginning of the study, as outlined in Table 1. The tool uses patient-focused parameters, including age, sex, body mass index (BMI) and VR-12 survey, to predict the likelihood of improvement after TKA. The predicted outcome is displayed in a format that is similar to the *After My Surgery* tool from the University of York [28]. This format uses a patient-friendly output of how 100 patients who were similar to the user felt after TKA surgery (Fig. 1). All other aspects of the study for participants in the intervention group will be identical to the TAU group.

Participants in the TAU group will receive standard care for TKA consideration without *SMART Choice* tool use. This includes clinic appointments, education packages, and booking/performance of TKA if clinically appropriate. TAU care will vary between institutions.

### Outcomes

The primary outcome for this study is the impact of the *SMART Choice* tool on patient willingness to undergo surgery. This will be measured using a binary question asking the patient if they would be willing to have TKA due to their knee symptoms, as described in Table 2.

**Table 2:** Schedule of Assessments (SoA)

	SCHEDULE OF ASSESSMENTS						
	Enrolment	Allocation to intervention	Post-allocation				Close-out
**TIME POINT**	***t***_***x***_	**t**_**0**_	***t***_***1***_	***t***_***2***_	***t***_***3***_	***t***_***4***_	***t***_***y***_
**ENROLMENT:**							
**Eligibility screen**	X						
**Informed consent**	X						
**Allocation to intervention**		X					
**ALLOCATION GROUPS:**							
**Intervention group (I)**		X					
**Treatment as usual group (TAU)**		X					
**INTERVENTION:**							
**Prognostic Tool Use**			I				
**ASSESSMENTS:**							
**Baseline questionnaire**		X					
**Willingness for surgery**		X		X	X	X	X
**Already proceeded with surgery**				X	X	X	X
**Qualitative questionnaires **						X	X

See also Table 3 for definitions and timepoints. I = intervention group. TAU = treatment as usual group. X = participants in both intervention and treatment as usual groups

The secondary outcomes for this study include:

- Determining the optimal timepoint for prognostic tool use in a patient’s TKA journey to maximise the effect on willingness to undergo surgery.
- Determine whether there are differences in the effectiveness of the *SMART Choice* tool when used in subpopulations such as sex, gender, and ethnicity.
- Understanding the user experience and effect on decision making of the *SMART Choice* tool through decision quality questionnaires.

### Participant timeline and assessments

**Fig. 2:**
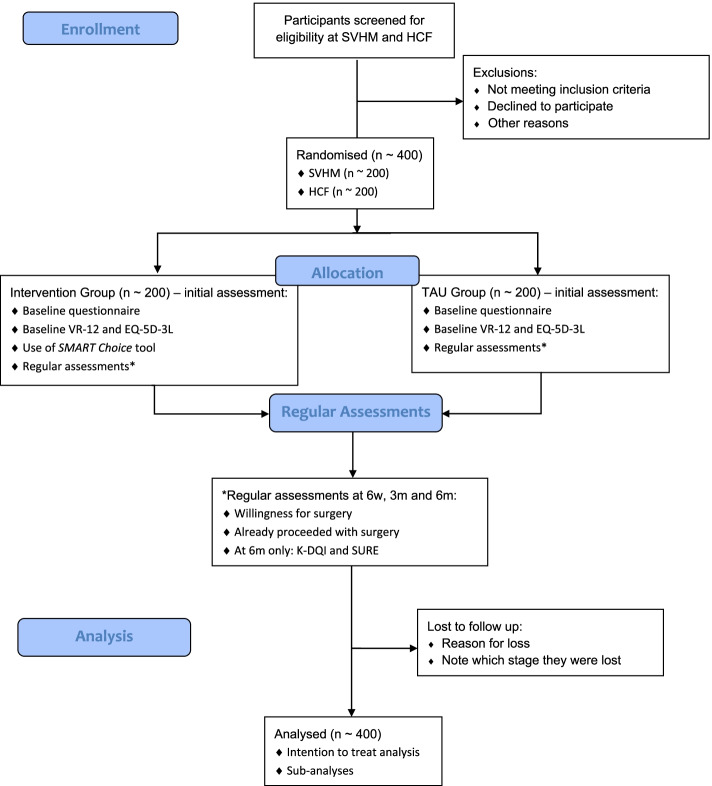
Flow diagram describing the study procedure. SVHM: St. Vincent’s Hospital, Melbourne; HCF: Hospitals Contribution Fund Australia; VR-12: Veterans RAND-12; EQ-5D-3L: EuroQol 5 Dimensions 3 Levels; TAU: treatment as usual; K-DQI: Knee Decision Quality Instrument; SURE: decisional conflict screening tool

Participants who meet the eligibility criteria will be contacted by the research team via email (Fig. 2). This email will contain a unique link to the study portal and participant access details, including participant username and password. On the study portal, participants will be able to read an electronic copy of the participant information sheet and consent form (PICF) and electronically sign the agreement to participate in the study. Once the PICF is signed, the participant is formally recruited to be part of the study. The study portal will have an in-built function to randomly allocate participants to an arm of the study: intervention or TAU group.

All recruited participants will be asked to complete a baseline questionnaire within the study portal. This provides an understanding of the participant’s general characteristics. The questionnaire will capture contact details, date of birth, sex, height, weight, comorbidities, medications (analgesia), smoking status, time with knee OA symptoms (years), previous nonoperative management of affected knee OA symptoms, time with knee OA symptoms, consultation with an orthopaedic surgeon in the past, appointment to see an orthopaedic surgeon arranged, previous surgery, previous injury to the affected knee, and presence of contralateral TKA. The baseline questionnaire will also capture the VR-12 and the Euroqol 5 Dimensions 3 Levels (EQ-5D-3L) data to understand the baseline HRQoL utility score for each group.

For participants in the intervention group, the study portal will allow access to the *SMART Choice* tool and provide a predicted outcome for the participant. Inputs onto the tool will be captured electronically; however, the participant will have the option to download a copy of their results for personal reference. Participants in the TAU group will not have access to the *SMART Choice* tool or predicted outcome and will instead continue with the regular assessments for the remainder of the study.

Following the baseline questionnaire (or *SMART Choice* tool use for the intervention group), participants will be asked to complete regular assessments in accordance with the Schedule of Assessments (Tables 2 and 3). This will consist of “willingness for surgery”, “already proceeded with surgery”, and decision quality questionnaires.

**Table 3:** Definitions for SoA

Terms	Definitions	
Time Point	***t***_***x***_	Time at initial contact via email with PIS, CF, and eligibiility screen. If a patient is eligible and consents, allocation will immediately follow this time.
	**t**_**0**_	Time at allocation with initial concurrent assessments.
	***t***_***1***_	Immediately after allocation, for prognostic tool use in TAU group.
	***t***_***2***_	6 weeks after initial assessment (t_0_)
	***t***_***3***_	12 weeks after initial assessment (t_0_)
	***t***_***4***_	6 months after initial assessment (t_0_)
	***t***_***y***_	Time at study closure for individual participant. This should be equal to t_4_ if all assessments are completed on schedule.
Baseline Questionnaire	Questionnaire for all patients who have been allocated in the study. Same for all groups. Captures basic demographic data as well as questions about previous knee treatment and surgery. This section will also capture baseline health-related quality of life through the VR-12 and EQ-5D-3L.	
Wilingness for Surgery	This assessment asks the question "Are your knee symptoms so bothersome that you wish to undergo surgery if medically fit to do so?" Yes / No. If yes, "In what time frame are you willing to have surgery?" [Time in months].	
Already Proceeded with Surgery	This assessment asks the question "Have you already received a TKA for your knee symptoms?" Yes / No. The purpose for this assessment is to 1) check if the patient has undergone TKA, and 2) outcome aligns with their willingness for surgery response.	
Qualitative Questionnaires	This assessment consists of K-DQI (decision quality) and SURE (decisional conflict) tools.	

*PIS* participant information sheet, CF consent form, *TKA* total knee arthroplasty, *OA* osteoarthritis, *VR-12* Veterans-RAND 12, *EQ-5D-3L* EuroQol 5 Dimensions 3 Levels, *K-DQI* Knee Decision Quality Instrument, *SURE* decisional conflict screening tool

The initial assessment will be the first timepoint at which regular assessments will be performed. At the end of the initial assessment, participants will be thanked for their time and be reminded of follow-up. Participants will receive emails with access to the study portal at subsequent follow-up periods: 6 weeks, 3 months, and 6 months after recruitment.

Willingness for surgery assessment consists of a single binary question: “Are your knee symptoms so bothersome that you would be willing to undergo surgery if medically fit to do so? (Yes/No)” If yes, “In what time frame are you willing to have surgery?” [Time in months]. The purpose of this assessment is to evaluate whether the patient is still of the mindset to proceed with surgery.

Already proceeded with surgery assessment consists of a single binary question: “Have you already received a TKA for your knee symptoms? (Yes/No).” The purpose of this assessment is to 1) correlate true outcomes for willingness for surgery if applicable and 2) differentiate baseline HRQoL data between knee OA symptoms (if not proceeded with surgery yet) or TKA outcomes. This will not be asked at the initial assessment.

All participants in the intervention group will be sent two additional decision quality questionnaires via email at the time of their final assessment (6 months after recruitment). These questionnaires have been validated from previous research to produce discriminatory and reproducible results [29, 30]. The addition of these questionnaires will provide a cross-sectional understanding of how useful the *SMART Choice* tool was for patient decision-making.

The first questionnaire is the “Knee Osteoarthritis Decision Quality Instrument” (K-DQI) [29]. This questionnaire is specific for patients who suffer from knee osteoarthritis. The questionnaire aims to assess:

- Which aspects of decision-making matter most to the patient
- How well the patient is understanding the information provided, and
- The level of communication between the patient and clinician prior to decision-making.

The second questionnaire is a short screening tool to assess decisional conflict [30]. It consists of four binary items using the acronym “SURE” (Table 4).

**Table 4:**
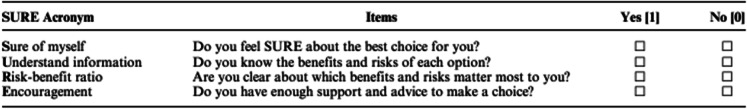
Binary items used in the SURE screening tool to assess decisional conflict

If a patient answers “no” to 1 or more questions, then the screen is considered positive for decisional conflict. Understanding decisional conflict is important to ensure that the information provided by *SMART Choice* is presented with clarity and aids the overall experience for patients on their TKA journey.

All participants will have free access to the *SMART Choice* tool at the completion of the study period.

### Sample size and recruitment

Previous studies have estimated baseline willingness for surgery in TKA candidates to be approximately 70% [31]. With the use of first-line interventions, such as therapy groups, willingness to undergo surgery was reported to decrease by 35% at 12 months [32]. We expect our cohort to have a lower baseline willingness for surgery, considering that we will have a significant proportion of participants recruited at the earlier (and less severe) stage of knee OA. We also expect our tool to have a greater impact on willingness to undergo surgery, especially for participants who were earlier in the TKA journey. To detect an absolute change in willingness to undergo surgery from 65 to 50%, a sample size of 169 participants in each arm was required based on calculations using a two-sided t-test at the 5% level of significance with 80% power. To account for a higher-than-expected loss to follow-up proportion due to the online nature of the tool, a 20% inflation of the sample size was added. This resulted in a target enrolment of 200 participants in each arm for a total sample size of 400 participants.

**Fig. 3:**
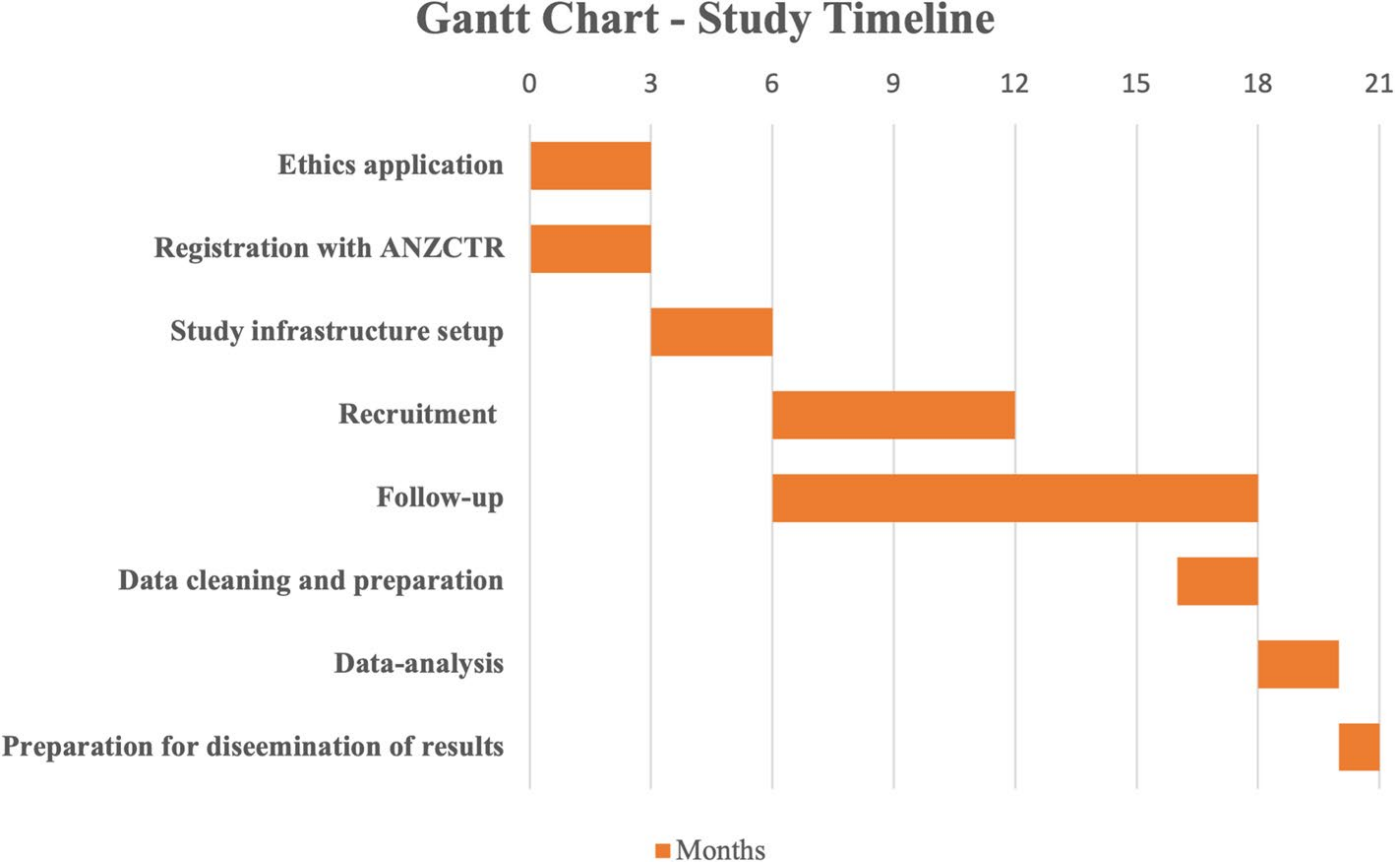
Gantt Chart detailing the timeline of the study. ANZCTR: Australian and New Zealand Clinical Trials Registration

The sample size calculated is a feasible number to recruit because SVHM performs approximately 400 TKAs each year, and HCF has approximately 50,000 members with knee OA. We will aim to recruit 200 patients from the SVHM cohort and approach a random pool of 200 patients with knee OA from the HCF client database. Based on these calculations, we estimate that 6 months will be sufficient time for recruitment to be completed, with a total study time estimate to be 21 months (Fig. 3).

### Data collection and management

The *SMART Choice* tool will be housed on a secure web-based platform accessible by participant account credentials only. Data captured from the *SMART Choice* tool will be linked with a participant account and deidentified. Regular assessments will be completed electronically via a secure online study portal using the same participant account credentials. All data will be entered electronically onto the database software REDCap (Research Electronic Data Capture). Hardcopy data for participants who require mail delivery will be entered manually onto REDCap by a research assistant. Access to the REDCap database will be restricted to researchers with staff credentials who are directly involved with the project. The data servers will be housed in secure facilities at SVHM and The University of Melbourne for participants recruited from SVHM and HCF, respectively. All data servers will be physically located in Australia to comply with local privacy laws and regulations. Hardcopy data will be kept in a secure facility with electronic swipe card access at SVHM Clinical Sciences Building. Wireless network access is encrypted using secure enterprise WPA2 encryption. All data will be retained for a period of 7 years from completion of the study and then destroyed permanently.

### Statistical plan

Willingness to undergo surgery will be evaluated by intention-to-treat analysis. Although there is no risk that participants in the TAU group will use the *SMART Choice* tool, there is a possibility that participants in the TAU group may seek alternative prognostic tools online. Limited disclosure in the consenting process will help to reduce participants who use alternative tools. Secondary analysis will assess the optimal timepoint for *SMART Choice* tool use. Data on the timepoint each participant is along the TKA journey will help facilitate this. Lost-to-follow-up participants will be included in the final analysis using multiple imputation methods. Categorical outcomes will be compared between groups using chi-squared tests. Relative differences in proportions between the intervention and TAU groups will be calculated as a relative risk with 95% confidence intervals. If there was an imbalance in variables associated with willingness and unwillingness to undergo surgery, logistic regression will be used to assess dichotomous outcomes and reported as odds ratios with 95% confidence intervals. Analysis of continuous outcomes will be performed with 2-sample and 2-tailed Student’s t-tests. Similarly, if there is an imbalance in variables associated with willingness and unwillingness to undergo surgery, continuous outcomes will be assessed using linear regression. We will employ a research assistant to support a high follow-up proportion of patients with respect to completing regular assessments, with a feasibility target of greater than 85%. All statistical analyses and model building will use RStudio software with the “tidyverse” packages.

### Ethics, registration and dissemination

This study has been approved by the Human Research Ethics Committees at both St. Vincent’s Hospitals, Australia (HREC 285/21), and The University of Melbourne (2021–23,157–24,025-2). This includes the questionnaire data for *SMART Choice* user experience. The University of Melbourne ethics approval pertains to participants recruited from the HCF cohort. Modifications to the protocol that impact the study procedure or analysis will be amended on both the protocol and the ethics application. The study has also been prospectively registered with the Australian and New Zealand Clinical Trials Registry (ACTRN12622000072718). There are no restrictions on the dissemination of results for this study, apart from acknowledgement of funding from the HCF Research Foundation. We plan to disseminate the results through peer-reviewed publications and conference presentations.

## Discussion

*SMART Choice* is not the first prognostic tool to be developed for use in TKA [33, 34]. However, to our knowledge, this will only be the second clinical trial evaluating the effect of a prognostic tool on patient decision making in TKA [35]. This is an important milestone in the implementation of such prognostic tools in clinical practice. A significant barrier to widespread prognostic tool use is the lack of data validating the performance and influencing these tools on patients in a pragmatic setting. By the nature of prognostic tool development, validation is often limited to internal subsets used to test the predictive model. The results from this trial will inform the arthroplasty community with evidence to support or refute the use of the *SMART Choice* tool in clinical practice.

We developed the *SMART Choice* tool with a patient focus in mind. Alternative prognostic tools have historically skewed towards the surgeon or clinician as the preferred user [34, 36–44]. This included predictors that were difficult for patients to input, such as knee range of motion, Kellgren-Lawrence score for X-ray findings, and blood markers. The *SMART Choice* tool aims to simplify the predictive variables to those a patient can input without clinician assistance. The rationale behind this design decision was based on our hypothesis that earlier use of prognostic tools in the TKA journey resulted in more influence on patient decision making. However, a major limitation of our prognostic tool design is the elimination of surgical and clinical factors that may have a bearing on the overall predictive outcome for TKA.

There are other important limitations to this study. Alternative prognostic tools for TKA, such as *After My Surgery,* are freely available for use on the internet [28]. Through participation in the study, participants may be incentivised to use alternative prognostic tools to compare their TKA outcome predictions. Although limited disclosure consent will be used to minimise the risk for this, we expect some participants to still access alternative prognostic tools. The consequence of this practice may present as difficulty isolating the results of this trial to the *SMART Choice* tool. An additional limitation stems from the expectation that participants who are considering TKA are generally in the older age category. The heavy reliance on technology in this study using study portals and online *SMART Choice* tool platforms may be seen as a barrier to participation. Nonetheless, we maintain an online approach for this study, as it reflects the pragmatic environment within which future usage of these tools are likely to exist. In addition, the decision quality questionnaires of the study will help us understand the user experience and how the tool works to change patient expectations around surgery. Furthermore, as a nested qualitative study, we will conduct semi-structured interviews to evaluate the patient experience when using the *SMART Choice* tool. Consequently, this will aid improvements in future versions of the tool.

In summary, we believe our study sets the framework for transitioning the *SMART Choice* tool as an academic body of work into a clinically usable prognostic tool. With the shifting paradigm in surgery towards “individualised care”, the *SMART Choice* tool aims to provide patients with an individualised prediction for TKA outcome. With the use of this tool, the delicate balance of surgical decision-making swings towards patients who can make more informed decisions about their own healthcare needs. Concurrently, clinicians can use this tool to help patients readjust their expectations prior to surgery. Future work in the field of predictive technologies will likely require a high level of scrutiny through clinical trials. Our study is an early step towards progressing the *SMART Choice* tool to be implemented in routine clinical practice.

## Data Availability

Not applicable.

## Abbreviations

ANZCTR: Australian and New Zealand Clinical Trials Registry
BMI: Body mass index
EQ-5D-3L: Euroqol 5-dimensional 3-level survey
GP: General practitioner
HCF: Hospital Contributions Fund (private health insurance company)
HRQoL: Health related quality of life
K-DQI: Knee Decision Quality Instrument
MCID: Minimal clinically important difference
OA: Osteoarthritis
PICF: Participant information sheet and consent form
PROM: Patient reported outcome measure
REDCap: Research Electronic Data Capture software
SMART: St. Vincent’s Melbourne Arthroplasty Outcomes (Registry)
SoA: Schedule of Assessments
SPIRIT: Standard Protocol Items for Randomised Trials
SURE: Decisional conflict screening tool
SVHM: St. Vincent’s Hospital, Melbourne
TAU: Treatment as usual
TKA: Total knee arthroplasty
TRIPOD: Transparent Reporting of Multivariable Prediction Model for Individual Prognosis or Diagnosis
VR-12: Veterans-Rand 12 Survey
WPA2: Wi-fi Protected Access v2
